# Spatial and temporal correlation in progressive degeneration of neurons and astrocytes in contusion-induced spinal cord injury

**DOI:** 10.1186/1742-2094-9-100

**Published:** 2012-05-25

**Authors:** Kyoung-Jin Min, Hey-Kyeong Jeong, Beomsue Kim, Dong Hoon Hwang, Hae Young Shin, An Tran Nguyen, Jong-hyeon Kim, Ilo Jou, Byung G Kim, Eun-hye Joe

**Affiliations:** 1 Department of Pharmacology, Ajou University School of Medicine, san-5 Woncheon-dong Youngtong-gu, Suwon, Kyunggi-do, 442-721, Korea; 2 Neuroscience Graduate Program, Ajou University School of Medicine, Suwon, Korea; 3 Institute for Medical Sciences, Ajou University School of Medicine, Suwon, Korea; 4 Brain Disease Research Center, Ajou University School of Medicine, Suwon, Korea; 5 Chronic Inflammatory Disease Research Center, Ajou University School of Medicine, Suwon, 442-721, Korea

**Keywords:** Microglia, Monocytes, Astrocytes, Spinal cord injury, Secondary injury

## Abstract

****Background**:**

Traumatic spinal cord injury (SCI) causes acute neuronal death followed by delayed secondary neuronal damage. However, little is known about how microenvironment regulating cells such as microglia, astrocytes, and blood inflammatory cells behave in early SCI states and how they contribute to delayed neuronal death.

****Methods**:**

We analyzed the behavior of neurons and microenvironment regulating cells using a contusion-induced SCI model, examining early (3–6 h) to late times (14 d) after the injury.

****Results**:**

At the penumbra region close to the damaged core (P1) neurons and astrocytes underwent death in a similar spatial and temporal pattern: both neurons and astrocytes died in the medial and ventral regions of the gray matter between 12 to 24 h after SCI. Furthermore, mRNA and protein levels of transporters of glutamate (GLT-1) and potassium (Kir4.1), functional markers of astrocytes, decreased at about the times that delayed neuronal death occurred. However, at P1 region, ramified Iba-1^+^ resident microglia died earlier (3 to 6 h) than neurons (12 to 24 h), and at the penumbra region farther from the damaged core (P2), neurons were healthy where microglia were morphologically activated. In addition, round Iba-1/CD45-double positive monocyte-like cells appeared after neurons had died, and expressed phagocytic markers, including mannose receptors, but rarely expressed proinflammatory mediators.

****Conclusion**:**

Loss of astrocyte function may be more critical for delayed neuronal death than microglial activation and monocyte infiltration.

## **Background**

In spinal cord injury (SCI), the primary damage is followed by secondary damage that results in expansion of the damaged area. Microenvironmental changes in the spinal cord caused by primary damage can induce secondary damage [1,2]. Although resident microglia and astrocytes, as well as blood leukocytes that infiltrate the injured spinal cord, can be an important regulator of the microenvironment, little is known about how these microenvironment-regulating cells behave in early injury states in response to SCI and whether they affect delayed neuronal death.

Microglia, as resident immune cells of the brain and spinal cord, scan their microenvironment and act as sensors of pathological changes [3]. For several decades, it has been generally accepted that, in response to injury or inflammatory stimuli, microglia are activated and produce cytotoxic proinflammatory mediators, such as tumor necrosis factor (TNF)-α, inducible nitric oxide synthase (iNOS), and reactive oxygen species (ROS) [4-6]. However, recent studies of injured brains and spinal cords have reported that microglia produce neuroprotective factors and increase phagocytic activity [7,8]. We also have reported that microglia do not contribute to delayed neuronal death associated with lipopolysaccharide (LPS)- or ATP-induced brain damage [9,10].

Astrocytes, the most abundant cells in the central nervous system (CNS), are important microenvironment-regulating cells. Astrocytes provide neurons with glucose and neurotrophic factors, and protect neurons from oxidative stress and excitotoxicity through glutathione (GSH) production, glutamate and potassium uptake, and modulation of water content [11-17]. Accordingly, it has been reported that ablation of astrocytes augments traumatic neuronal damage, and that transplantation of astrocytes diminishes brain damage [18,19].

Blood leukocytes, including neutrophils and monocytes, are other important players in the CNS inflammation. Neutrophils infiltrate injured brain and spinal cord regions, and produce neurotoxic effects by promoting the expression of iNOS, cyclooxygenase (COX)-2, and several cytokines [10,20,21]. Monocytes infiltrate injured brain and spinal cord regions. For monocytes, however, dual roles have been reported: promoting clearance of the injured area [22] and producing neurotoxic factors [9,23,24].

The aim of this study was to understand the discrete roles of microenvironment-regulating cells in delayed neuronal injury. Interestingly, we found that ramified Iba-1^+^ cells (resident microglia) died earlier than neurons in the area where delayed neuronal death occurred, and that neurons were healthy where microglia were morphologically activated. Round Iba-1^+^ cells that highly expressed CD45 appeared after neurons had died, and expressed phagocytic activity. Interestingly, the spatial and temporal patterns of astrocyte and neuron death were similar. Therefore, we suggest that Iba-1^+^ cells, including ramified and round cells, are innocent in delayed neuronal death, and speculate that loss of the supportive function of astrocytes may contribute to delayed neuronal death.

## **Materials and methods**

### **Ethics statement**

All experiments were performed in accordance with the approved animal protocols and guidelines established by the Ajou University School of Medicine Ethics Review Committee, and all animal work was approved by the Ethical Committee for Animal Research of Ajou University (Amc-28).

### **Preparation of contusion-induced SCI**

As previously described [25], female Sprague–Dawley rats (200 to 250 g) were anesthetized using chloral hydrate (360 mg/kg, i.p.), and a dorsal laminectomy was performed at the ninth thoracic vertebra level (T9) to expose the spinal cord. A mechanical force (150 kdyn, weight diameter: 2 mm) was applied to the exposed spinal cord using the Infinite Horizon Impactor (Precision Systems and Instrumentation, Lexington, KY, USA). Rats that received laminectomy alone were used as sham-operated controls. Prior to sacrificing, from 3h to 14 d after surgery, rats received postoperative care that included manual excretion of urine twice daily until bladder function returned.

### **Tissue preparation**

**Figure 1:**
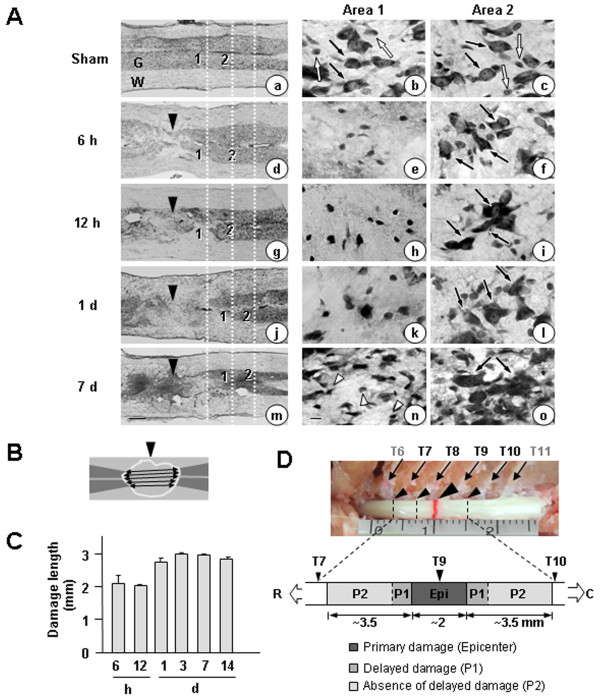
**Time-dependent gray matter damage in the contusion-induced SCI model.** Contusion insult (150 kdyn) was applied to T9 of rat spinal cords. Longitudinal sections (30 μm) were obtained from 6 h to 14 d after the injury. **(A)** Sections were stained with cresyl violet. Sham-operated animals were employed as controls. In left panels, gray matter (G) was intensely stained with cresyl violet (compare to white matter, W). Right panels (Area 1 and Area 2) are higher magnifications of Areas 1 and 2 indicated in the left panels. Large (black arrows) and small (white arrows) Nissl^+^ cells are neurons and glial cells, respectively. At 7 d, many small Nissl^+^ cells (white arrowheads) appeared. Dotted white lines in the left panel show the positions of Area 1 and Area 2 relative to the contusion sites. Black arrowheads: contusion sites. **(B)** The most damaged cresyl violet-stained sections obtained at the indicated times were selected, and the extent of damage was evaluated by measuring the length of gray matter, as described in Materials and Methods. Black arrowheads: contusion sites. **(C)** Graphic presentation of the values measured in (B). Values in (C) are means ± SEMs of four to six animals. **(D)** Relative positions of contusion site (arrowhead at T9), primary damage site (epicenter), delayed damage-occurring site (penumbra 1), and delayed damage-absent site (penumbra 2). Spinal cords between T7 and T10 were removed and analyzed. Scale bars: 500 μm (left panel in A) and 25 μm (right panel in A). Data shown are representative of three independent experiments. In each experiment, four to six animals were used for each time point.

Rats were anesthetized and transcardially perfused with a saline solution containing 0.5% sodium nitrate and heparin (10 U/ml), and then with 4% paraformaldehyde in 0.1 M phosphate buffer (PB) at pH 7.4 to fix tissues. Spinal cords from T7 to T10 (about 9 to 11 mm length) were removed (Figure 1D), post-fixed overnight at 4°C in 4% paraformaldehyde, and stored at 4°C in a 30% sucrose solution until they sank.

Longitudinal sections (30 μm thickness) and cross sections (30 μm thickness) of spinal cords were obtained using a cryostat (Leica, Wetzlar, Germany). More than 300 cross sections were obtained from a spinal cord. Sections were attached to 30 to 35 slides in the way the first section was attached to the first slide, and the second slide was attached to the second slide, and so on. In this way, each slides included 9 to 12 sections. To examine behavior of neurons, astrocytes and microglia, three serial slides that included immediately adjacent cross sections were stained with antibodies specific for NeuN (a neuron marker), GFAP (an astrocyte marker), and Iba-1 (a microglia/monocyte marker) (Additional file 1: Figure S1).

For reverse transcription-polymerase chain reaction (RT-PCR), quantitative real-time PCR (q-PCR) and Western blot analysis, spinal cord tissues (approximately 4 mm total length around the contusion center) were obtained without perfusion with 4% paraformaldehyde.

### **Measurement of damage areas**

Longitudinal sections were stained with cresyl violet, a Nissl stain. Stained sections were photographed using an Olympus BX51 microscopy camera (Olympus, Ballerup, Denmark). The damaged area was defined as the area in which large Nissl^+^ neurons disappeared, presented as lesion length [26,27]. Because the width of gray matter in spinal cords varied among animals, it was more accurate to measure length than area. To measure lesion length using the public domain JAVA image-processing program IMAGE J (http://rsb.info.nih.gov/ij), we selected the horizontal section containing the maximum lesion, drew five lines from the top to the bottom of the gray matter, and measured the distance of each line between the rostral and the caudal area where Nissl^+^ neurons had disappeared as shown in Figure 1B.

### **Immunohistochemistry**

For 3,3'-diaminobenzidine (DAB) staining, sections were rinsed three times in phosphate-buffered saline (PBS) and pretreated for five minutes with PBS containing 3% H_2_O_2_. They were then rinsed with PBS containing 0.2% Triton X-100 (PBST), blocked with PBST containing 1% bovine serum albumen (BSA) for 30 minites, and incubated for 2 h at room temperature with primary antibodies against CD45 (1:200; AbD Serotec, Oxford, UK), CD68 (1:200; AbD Serotec), EAAT2 (GLT-1, 1:500; Abcam, Cambridge, MA, USA), glial fibrillary acidic protein (GFAP, 1:300; Sigma, St. Louis, MO, USA), IL-1β (1:200; R&D Systems, Minneapolis, MN, USA), iNOS (1:200; abcam), ionized calcium binding adaptor molecule 1 (Iba-1, 1:1,000; Wako Pure Chemical Industries, Osaka, Japan), Kir4.1 (1:200; Alomone Labs, Jerusalem, Israel), mannose receptor (MR, 1:50; Abcam), or neuronal nuclei (NeuN, 1:300; Chemicon, Temecula, CA, USA). Sections were then rinsed in PBST, incubated with biotinylated secondary antibodies (Vector Laboratories, Burlingame, CA, USA), visualized using 0.05% DAB and 0.003% hydrogen peroxide in 0.1 M PB, and examined under a bright-field microscope (Olympus Optical, Tokyo, Japan). For double-immunofluorescence staining, sections were dried for 1 h, washed twice in PBS, treated with 1% BSA for 30 minutes, and then incubated with the appropriate combination of antibodies against NeuN, Iba-1, GFAP, iNOS, CD45, CD68 and mannose receptor. Immunoreactive proteins were visualized using Alexa Fluor 488- or Alexa Fluor 555-conjugated secondary antibodies (1:600 dilution; Invitrogen, Eugene, OR, USA). Cells were counterstained with 4',6-diamidino-2-phenylindole (DAPI; Vector Laboratories) to detect nuclei. Images were analyzed under a confocal microscope (LSM 510, Carl Zeiss, Jena, Germany) or a florescence microscope (Carl Zeiss). For cresyl violet (Sigma) staining, sections were rehydrated, placed in cresyl violet for 15 to 20 minutes, then rinsed and dehydrated with a series of graded alcohols (70% to 100%). Sections were placed in xylene and mounted.

### **Terminal deoxynucleotidyl transferase-mediated deoxyuridine triphosphate nick-end labeling (TUNEL) assays**

TUNEL assays were performed using the Apoptag Fluorescein *In Situ* Detection Kit (Intergen, Purchase, NY, USA) according to the manufacturer’s instructions. Briefly, spinal cord sections were post-fixed in pre-cooled permeabilization solution (ethanol:acetic acid, 2:1) for five minutes and incubated with TUNEL reaction mixture in a humidified chamber at 37°C for one hour. After washing with PBS, sections were incubated with anti-Iba-1 antibody for two hours, washed in PBS, and then incubated with Texas Red-labeled secondary antibodies.

### **RT-PCR and q-PCR**

Spinal cord tissues were dissected, after which total RNA was isolated using RNAzol B (iNtRON, Sungnam, Korea) and cDNA was prepared using Avian Myeloblastosis Virus reverse transcriptase (Promega, Madison, WI, USA) according to the manufacturer’s instructions using a Corbett thermal cycler (Corbett Research, Sydney, NSW, Australia). PCR was performed in a final volume of 25 μl using HiQ Taq Red DNA Polymerase (GenDEPOT, Barker, TX, USA) and a Corbett thermal cycler. The amplified products were separated by electrophoresis on a 1.0% agarose gel, and detected under UV light. For q-PCR, cDNA and forward/reverse primers (200 nM) were added to 2x KAPA SYBR Fast Master Mix and reactions were performed on an RG-6000 real-time amplification instrument (Corbett Research). The threshold cycle number (Ct) of each target was calculated and expressed relative to that of GAPDH (glyceraldehyde-3-phosphate dehydrogenase), used as a reference. Delta-delta Ct values of targets were presented as relative fold induction. Primers (Bioneer, Daejeon, Korea) used in q-PCR/RT-PCR are shown in Table 1.

**Table 1:** Primer sequences for RT-PCR and/or qPCR

Gene	Sense	Antisense
IL-1	5′-TGATGTTCCCATTAGACAGC-3’	5′-GAGGTGCTGATGTACCAGTT-3’
IL-6	5′-AAAATCTGCTCTGGTCTTCTGG-3’	5′-GGTTTGCCGAGTAGACCTCA-3’
COX-2	5′-ACACTCTATCACTGGCATCC-3’	5′-GAAGGGACACCCTTTCACAT-3’
iNOS	5′-GCAGAATGTGACCATCATGG-3’	5′-ACAACCTTGGTGTTGAAGGC-3’
GLT-1	5′-GGCACCTTTCTTCAGTCAGC-3’	5′-GCTTTCAACTGGTCTCAGGC-3’
Kir4.1	5′-CACAGCTCCGCTCGCCACTC -3’	5′-AGGGGCCGGCTCTCTGTCTG-3’
GAPDH	5′-TCCCTCAAGATTGTCAGCAA-3’	5′-AGATCCACAACGGATACATT-3’

### **Western blot analysis**

Spinal cord tissues were dissected and homogenized in modified RIPA buffer (50 mM Tris–HCl pH 7.4, 1% NP-40, 0.25% Na-deoxycholate, 150 mM NaCl, 1 mM Na_3_VO_4_, 1 mM NaF) containing protease inhibitors (2 mM phenylmethylsulfonyl fluoride, 10 μg/ml leupeptin, 10 μg/ml pepstatin, 2 mM EDTA). Each lysate was centrifuged at 10,000 x g for 10 minutes at 4°C, and the supernatants were collected. Proteins were separated by SDS-PAGE, and transferred to a nitrocellulose membrane. The membrane was incubated with primary antibodies against iNOS (Upstate Biotechnology, Lake Placid, NY, USA), COX-2 (Santa Cruz Biotechnology, Santa Cruz, CA, USA), or actin (Santa Cruz Biotechnology), followed by incubation with horseradish peroxidase (HRP)-conjugated secondary antibodies (Zymed Laboratories, San Francisco, CA, USA) and visualization using an enhanced chemiluminescence system.

### **Statistical analysis**

Comparisons between two groups were analyzed using Student’s *t*-test. Comparisons among multiple groups were analyzed by one-way ANOVA followed by *post hoc* Student–Newman–Keuls tests using the Statistical Package for Social Sciences 8.0 (SPSS Inc., Chicago, IL, USA).

## **Results**

### **Microglia die earlier than neurons and astrocytes in the penumbra region in SCI**

In spinal cord injury (SCI), the primary damage is followed by delayed secondary damage. However, it is still ambiguous what causes delayed damage. Although inflammation has been suggested as a cause of delayed neuronal death in SCI, emerging evidence has suggested that inflammation in acute injury also exerts beneficial effects [28,29]. To address the cause of delayed neuronal death and the controversy surrounding the role of inflammation in the injured spinal cord, we analyzed the behavior of neurons and other microenvironment regulating cells using a contusion-induced SCI model, examining early (6 h) to late times (14 d) after the injury. Using Nissl staining, we first examined time-dependent neuronal damage in the gray matter of longitudinal sections of the spinal cord between T7 and T10 after the weight-drop at T9 (Figure 1A, D). In Figure 1A, right panels (Area 1 and Area 2) are higher magnifications of Areas 1 and 2 indicated in the left panels. Dotted white lines in the left panel show the positions of Area 1 and Area 2 relative to the contusion sites (Figure 1A). In sham-operated spinal cords, large Nissl^+^ neurons (black arrows in Figure 1Aa, b, c) and small Nissl^+^ glial cells (white arrows in Figure 1Aa, b, c) were detectable throughout the gray matter. Intact spinal cords showed a pattern similar to that of sham-operated spinal cords (data not shown). At areas near contusion sites (black arrowheads in Figure 1Ad, g, j, m), a loss of large Nissl^+^ cells was clearly evident within 6 h (Figure 1Ad, e). The neuron-damage area increased between 12 h and 1 d (Figure 1Ag vs. Figure 1Aj), but did not further increase thereafter until 7 to 14 d (Figure 1Aj vs. Figure 1Am; Figure 1C). Small Nissl^+^ cells detected at 7 d (white arrowheads in Figure 1An) appeared to be glia and/or infiltrated blood cells, as shown in the following studies. We determined neuron-damage areas by measuring the length of gray matter where large Nissl^+^ cells had disappeared at five positions evenly divided from top to bottom, as shown in Figure 1B. Since the extent and shape of gray matter collapse due to contusion injury varied among animals, we measured length instead of area. Using these measurements, we plotted the time-dependence of death in areas where neurons died (Figure 1C). Based on these measurements, we designated the contusion center (approximately 2 mm) as the epicenter where neurons acutely died directly due to the weight. In the penumbra region, the areas where neurons underwent death between 12 h and 1 d were designated as P1, and the areas where neurons were healthy for up to 14 d as P2 (Figure 1C, D).

**Figure 2:**
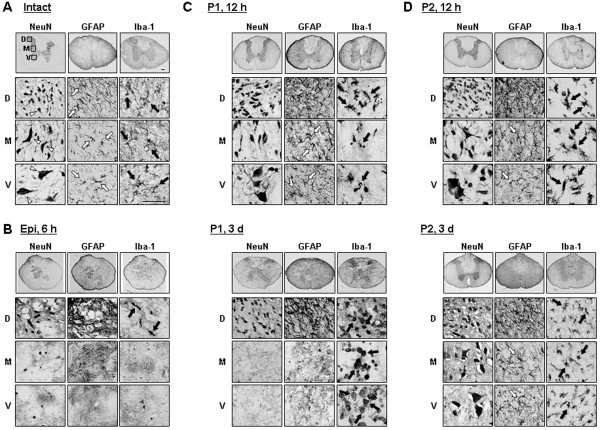
**Time-dependent behavior of neurons, resident microglia, and astrocytes in injured spinal cords.** Cross sections (30 μm) were obtained from the intact spinal cord **(A)**, epicenter **(**Epi, **B)**, P1 **(C)**, and P2 **(D)** areas in the caudal direction at the indicated times after injury. The behaviors of neurons (white arrowheads), astrocytes (white arrows), and resident microglia/monocytes (black arrows) were compared by staining immediately adjacent serial sections with antibodies against NeuN, GFAP, and Iba-1, respectively, and then visualizing using HRP-conjugated secondary antibodies. Dorsal (D), medial (M), and ventral (V) areas were magnified in the lower panels. Data shown are representative of more than three independent experiments. In each experiment, at least five animals were used for each time point. Scale bar: 200 μm (upper panels), 50 μm (lower panels).

Next, we further examined time-dependent behavior of neurons, astrocytes and microglia in immediately adjacent cross sections using antibodies specific for NeuN, GFAP, and Iba-1, respectively (Additional file 1: Figure S1). Because neurons in rostral and caudal areas showed similar delayed damage behavior (data not shown), we here show caudal areas at dorsal (D), medial (M), and ventral (V) areas (Figure 2). In intact spinal cords, healthy dorsal neurons appeared as small, round cell bodies and healthy ventral neurons appeared as large, polygonal cell bodies (white arrowheads in Figure 2A). Neurons in the medial region were larger than those in dorsal regions but smaller than those in the ventral region (white arrowheads in Figure 2A). GFAP^+^ astrocytes exhibited very different morphologies in intact dorsal, medial and ventral areas: in the dorsal area, astrocytes were dense and had highly branched processes, whereas astrocytes in the medial and ventral region had long, thin processes (white arrows in Figure 2A). Iba-1^+^ microglia, however, showed a highly ramified morphology that was similar in all dorsal, medial and ventral regions (black arrows in Figure 2A). Morphologies of neurons, microglia and astrocytes in sham-operated animals were the same as in intact animals (data not shown).

Immunostaining with specific markers of each type of cell showed that in response to SCI, neurons as well as astrocytes and microglia almost completely disappeared within 6 h after the contusion injury in the ventral and medial regions of the damage core (epicenter) (Figure 2B). However, in dorsal regions, neurons, astrocytes and microglia were healthy at 6 h (Figure 2B) and remained healthy at 7 d (data not shown), although Iba-1^+^ microglia lost their processes and exhibited an activated morphology (black arrows in Figure 2B). The different behaviors of neurons, astrocytes and microglia in dorsal and medial/ventral areas could reflect differences in mechanical forces applied to each area (ventral areas may function as a cushion for dorsal areas) and/or difference in the vulnerability of cells in each area.

**Figure 3:**
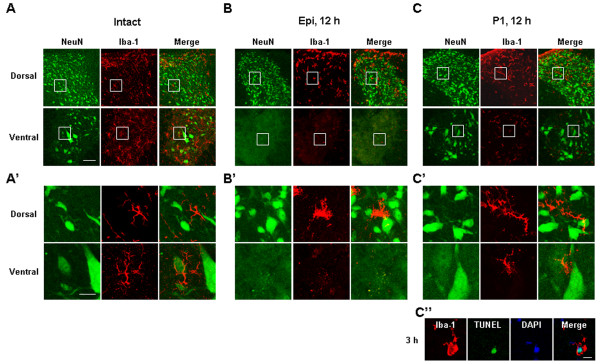
**Microglial behavior in SCI is not correlated with neuronal death.** Cross sections were obtained from the intact spinal cord **(A, A’)**, epicenter **(B, B’)**, and P1 regions **(C, C’, C”)** in the caudal direction at the indicated times after injury, and double-stained with anti-NeuN and anti-Iba-1 antibodies. NeuN and Iba-1 immunopositive regions were visualized with Alexa 488 (green)- and Alexa 555 (red)-conjugated secondary antibodies, respectively. (**A’-C’**) Higher magnification images of boxed regions in panels (A-C). (**C”**) Cross sections from the P1 regions were stained with TUNEL (green) and Iba-1 (red). Nuclei were counterstained with DAPI. Scale bars: 100 μm (A), 20 μm (A’) and 10 μm (**C”**). Data shown are representative of at least three independent experiments.

An interesting finding was the behavior of Iba-1^+^ microglia in the penumbra region close to the epicenter (P1 in Additional file 1: Figure S1B, and Figure 2C). At 12 h after SCI, Iba-1^+^ microglia in the medial and ventral areas were dead and those in the dorsal area were morphologically activated (black arrows in Figure 2C). Notably, neurons and astrocytes were still alive in all areas in the adjacent serial sections (Figure 2C), although astrocytes became hypertrophic (white arrows in Figure 2C). Double-labeling using anti-NeuN and anti-Iba-1 antibodies confirmed these findings. In intact spinal cords, neurons and microglia were healthy in all dorsal and ventral regions (Figure 3A, 3A’). In the epicenter, neurons and microglia in the ventral areas disappeared at 12 h, but were alive in the dorsal areas (Figure 3B, B’). In the penumbra regions close to the epicenter (P1), neurons in the dorsal and ventral areas were still intact at 12 h (Figure 3C, C’) although microglia in the ventral areas were not intact (Figure 3C, C’) as evidenced by TUNEL staining of ventral microglia at 3-6 h after SCI (Figure 3C”).

**Figure 4:**
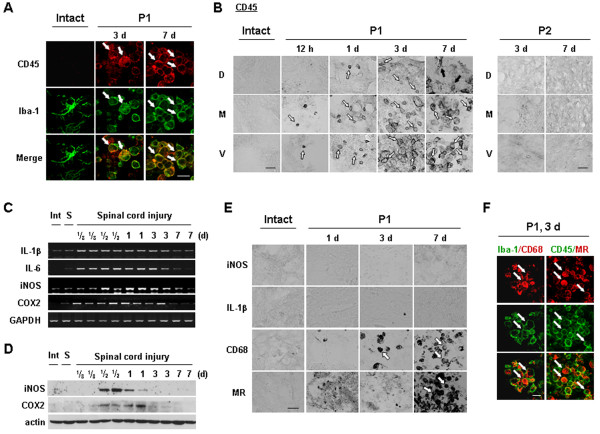
**Iba-1**^**+**^**/CD45**^**+**^**cells appear and express phagocytic activity in injured spinal cords. (A, B, E, F)** Sections from intact, P1, or P2 regions at the indicated times after the injury were labeled with indicated antibodies. White arrows indicated positively labeled cells with each antibody. (B) Black arrows (P1, 7 d) indicated CD45^+^ cells that are not round. (**C, D**) Spinal cord tissues were obtained at the indicated times after SCI, and total RNA and protein were prepared and analyzed by RT-PCR (**C**) and Western blotting (**D**), respectively, as described in Materials and methods. Data shown are representative of at least three independent experiments. Scale bars: 20 μm.

In analysis of cross sections obtained at 3 d, we could not find any serial sections where neurons and astrocytes were alive but microglia disappeared in medial and ventral areas, which was shown in P1 areas at 12 h (Additional file 1: Figure S1B, and Figure 2C). We further found that immunoreactivity of S100β (another astrocyte marker) in P1 ventral areas also remained at 12 h, but decreased at 3 d (Additional file 2: Figure S2). Therefore, neuron (and/or gray matter) and astrocyte damage increased between 12 h and 3 d (arrowheads in Additional file 1: Figure S1B) as shown in longitudinal sections (Figure 1). In addition, round Iba-1^+^ cells appeared in the medial and ventral areas where ramified resident microglia disappeared (black arrows in Figure 2C). In the dorsal areas, round and/or rod-shaped Iba-1^+^ cells were also detectable (black arrows in Figure 2C). In subsequent experiments, we found that these round Iba-1^+^ cells were labeled with CD45 unlikely ramified Iba-1^+^ cells in intact spinal cord (see Figure 4A).

**Figure 5:**
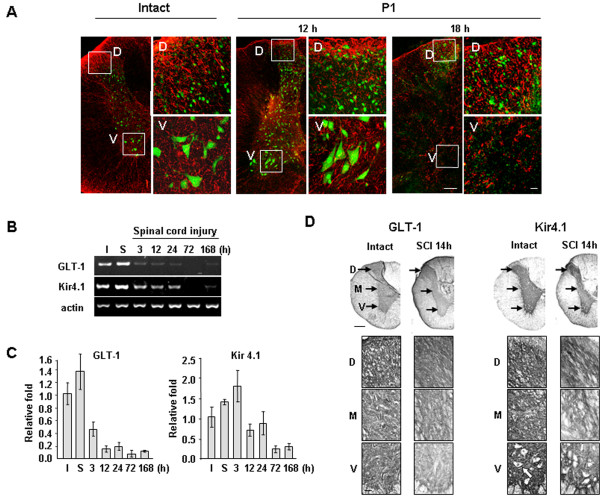
**Loss of astrocytes and reduced expression of GLT-1 and Kir4.1 in injured spinal cords. (A, D)** Sections from intact and P1 areas at the indicated times after injury were stained with NeuN/GFAP (A), and GLT-1 or Kir4.1 (**D**) antibodies. Right panels in (**A**) and lower panels in (**D**) are higher magnification images of indicated regions in the left and upper panels, respectively. **(B, C)** Spinal cord tissues (2 mm in length around the contusion center) were obtained at the indicated times after SCI, and total RNA was prepared and analyzed by RT-PCR (**B**) or q-PCR (**C**) as described in Materials and Methods. Values in (**C**) are means ± SEMs of five animals. (**D**) The white arrow represents neuronal cell body-like morphology. Data shown are representative of three independent experiments. Scale bars: 200 μm (left panels in A and upper panels in D), 10 μm (right panels in A), and 50 μm (lower panels in D).

It is also noteworthy that, in the P2 penumbra region farther from the epicenter than the P1 region, microglia were morphologically activated at 12 h and 3 d (black arrows in Figure 2D), but neurons and astrocytes were still healthy (Figure 2D). In the P2 region, astrocytes became transiently hypertrophic at 12 h (white arrows in Figure 2D). Thus, astrocytes at 3 d were similar to that in the intact spinal cord (white arrows in Figure 2D). Taken together, these results suggest that microglia are innocent in delayed neuronal death in SCI: microglia died earlier than neurons in the P1 region, and neurons were healthy in the P2 region where microglia were activated. Furthermore, it is noticeable that the time course of the disappearance of astrocytes in P1 ventral areas coincided with that of neuronal death in these areas. We further examined correlation between death of neurons and astrocytes in Figure 5.

### **Iba-1**^**+**^**/CD45**^**+**^**cells appear in injured spinal cord and exhibit phagocytic activity**

In P1 medial and ventral regions, round Iba-1^+^ cells were found at 3 d after SCI although ramified microglia died at 3 to 6 h (Figure 2C3 d vs. 12 h, and see Figure 3C”). In experiments to analyze the round Iba-1^+^ cells, we found that these cells expressed CD45, a marker of microglia and monocytes [30,31] unlike ramified resident microglia that expressed Iba-1 but not CD45 (see Figure 4A). The CD45^+^ cells were barely detectable at 12 h, and a few were found at 1 d (white arrows in Figure 4B). The numbers of CD45^+^ cells increased at 3 and 7 d (white arrows in Figure 4B). In dorsal areas, CD45^+^ cells were detectable, but there were fewer CD45^+^ cells in these areas at all time points than in ventral and medial areas (Figure 4B). In addition, at 7 d, CD45^+^ cells in the dorsal area had adopted a ramified morphology similar to that of activated microglia (black arrows in Figure 4B), whereas those in the ventral and medial areas maintained a round shape (white arrows in Figure 4B). In the epicenter (the damage core), CD45^+^ cells showed a behavior similar to that in P1 areas (data not shown). However, in the P2 region where neither neurons nor astrocytes were injured, CD45^+^ cells were not detectable at any time points (Figure 4B). Collectively, these results indicate that round Iba-1^+^/CD45^+^ cells appeared in the injured spinal cord and filled the area where ramified Iba-1^+^ resident microglia and neurons had died.

We next examined the expression of several inflammatory mediators in injured spinal cords. Interestingly, both mRNA and protein levels of the major proinflammatory mediators, IL-1β, IL-6, iNOS and COX-2, increased and then decreased before CD45^+^ cells appeared in the injured spinal cord. RT-PCR analysis indicated that IL-1β, IL-6, iNOS, and COX-2 mRNA levels were increased within 3 to 6 h after SCI, remained elevated for up to 1 d, and decreased at 3 d (Figure 4C). Protein expression of iNOS and COX-2 was also detectable between 12 h and 1 d (Figure 4D). Accordingly, neither iNOS nor IL-1β protein was detectable immunohistochemically in the gray matter of injured spinal cords at 1 to 7 d (Figure 4E). Although IL-1β protein was detected in morphologically activated microglia in the P2 area at 12 h (Additional file 3: Figure S3), delayed neuronal death did not occur in this area. Moreover, iNOS was detected in neutrophils at 1 d after SCI (Additional file 4: Figure S4) as previously described by others [32].

Next, we examined the possibility that these Iba-1^+^/CD45^+^ cells exhibited phagocytic activity (31). Therefore, we examined whether Iba-1^+^/CD45^+^ cells expressed CD68 and mannose receptor (MR) that are involved in the repair process by engulfing damaged cells and debris [33,34]. MR expression was detectable at 7 d (Figure 4E). CD68 expression was detectable at 3 d and further increased at 7 d. In double-labeling experiments using combinations of Iba-1/CD68 or CD45/MR antibodies (raised in different species), CD68 and MR immunoreactivity were detected in Iba-1^+^ and CD45^+^ cells, respectively (Figure 4F). Taken together, these results suggest that, instead of causing delayed neuronal death in SCI, Iba-1^+^/CD45^+^ cells function to clean up the damaged spinal cord.

### **The temporal and spatial patterns of astrocyte loss are coincident with those of neuron loss**

It is well known that astrocytes maintain the homeostasis of extracellular glutamate and potassium; they also scavenge ROS, produce growth factors and prevent excessive inflammation [35-40]. Since astrocytes disappeared in P1 medial and ventral areas at times similar to those when the disappearance of neurons was noted (Figure 2), we further evaluated the spatial and temporal relationship between the deaths of neurons and astrocytes by double-label immunohistochemistry using antibodies against NeuN and GFAP. In intact spinal cords, neurons and astrocytes were healthy in all dorsal and ventral regions (Figure 5A). As we expected, in the P1 region at 12 h after SCI, neurons and astrocytes were healthy in all dorsal and ventral areas (Figure 5A). However, in the P1 region at 18 h (at 18 to 24 h depending on animals), neurons and astrocytes became undetectable in ventral areas whereas both cell types were healthy in dorsal areas (Figure 5A).

We further examined time-dependent changes in the mRNA levels of transporters of glutamate (GLT-1) and potassium (Kir4.1) that are expressed in astrocytes [16,17]. SCI increases extracellular K^+^ and glutamate owing to the release of intracellular K^+^ and glutamate from damaged cells [41-43]. Therefore, surrounding neurons could be damaged if Kir4.1 and/or GLT-1 do not function properly [16,17,44,45]. RT-PCR and q-PCR revealed significant reductions in GLT-1 mRNA levels at 3 and 12 h after SCI, and Kir4.1 mRNA levels at 12 and 72 h after SCI (Figure 5B, C). In immunohistochemistry, similar results were obtained (Figure 5D). In intact spinal cords, GLT-1 was evenly expressed in the dorsal, medial, and ventral areas in the gray matter (Figure 5D: lower panels are higher magnifications of indicated areas in the upper panels). Kir4.1 also evenly expressed in the all areas in the gray matter except neuronal cell bodies (white arrow in Figure 5D). However, at 14 h after SCI, immunoreactivities of both GLT-1 and Kir4.1 were reduced in the medial and ventral areas while maintained in the dorsal areas (Figure 5D). These results suggest that loss of astrocytes and/or astrocyte function results in the loss of homeostasis of extracellular glutamate and potassium, which may contribute to delayed neuronal death.

## **Discussion**

The major findings in this study are as follows: First, there is no evident connection between microglial activation and delayed neuronal death. In the P1 region, microglia died earlier than neurons; and in the P2 region where microglia were morphologically activated, neurons were healthy. Second, Iba-1^+^/CD45^+^ cells appeared where neurons and microglia had died, and expressed phagocytic activity. Third, astrocyte death and/or loss of function occurred with a temporal and spatial pattern similar to that of delayed neuronal death.

It has been generally accepted that microglia are activated in brain/spinal cord injury and aggravate the injury. However, in previous studies, we and others have reported that inflammatory responses are very different between cultured microglia and microglia in the brain. Purified cultured microglia produce neurotoxic inflammatory mediators, such as iNOS and TNF-α, in response to inflammatory stimulators, including LPS, thrombin and interferon-γ [4-6]. However, in the LPS- or ATP-injected brain, and in the ischemic brain, microglia barely express these inflammatory mediators, and are more vulnerable to injury than are neurons [9,10,21]. This is partly due to the fact that, in the brain, astrocytes inhibit inflammatory responses [39,40]. Furthermore, cerebrospinal fluid is continuously produced and circulates [46], a process that removes endogenously produced inflammatory stimulators. In addition, recent studies have reported that activated microglia exert neuroprotective actions through production of neurotrophic factors and increase in phagocytic activity [28,29,47-49]. The results obtained in the current study also suggest that microglia do not play a cytotoxic role in delayed neuronal damage after SCI: microglia underwent death somewhat earlier than neurons in P1 ventral regions (Figures 2C, and 3C, C’ C”). Furthermore, at P1 dorsal and P2 dorsal/ventral regions, neurons were healthy, although they were surrounded by Iba-1^+^ cells with an activated morphology (Figures 2C, D and 3B C). Expression of the inflammatory mediators, IL-1β, IL-6, iNOS and/or COX-2, was detectable at the mRNA level 3 to 24 h following SCI, and at the protein level after 12 to 24 h (Figure 4C, D), as previously reported by others [50-52]. However, neurotoxic iNOS [53] was detected in neutrophils in damaged areas (Additional file 4: Figure S4). Although IL-1β was detected in morphologically activated microglia in the P2 region (Additional file 3: Figure S3), we have previously found that high levels of IL-1β expressed in microglia do not promote neurotoxicity [9]. Collectively, these observations suggest that microglia may not be neurotoxic in the injured spinal cord.

Several studies have reported that monocytes infiltrate from the blood to the injured spinal cord [54]. Iba-1 is highly expressed in both monocytes and microglia; these two types of cells can be discriminated on the basis of CD45 expression levels. CD45 is highly expressed in monocytes but only weakly in microglia [9,30,31]. Iba-1^+^/CD45^+^ cells were not detectable in intact spinal cords, but were detectable in P1 ventral and medial areas beginning at 12 h, increased at 1 d, and peaked at 3 and 7 d (Figure 4A, B), which is later than the times of neuronal death in P1 (Figures 2C and 4B). A more important finding is that these Iba-1^+^/CD45^+^ cells in P1 regions expressed CD68 and MR, indicators of phagocytic activity [33,34], rather than proinflammatory iNOS and IL-1β (Figure 4E, F). Although we could not exclude the possibility that ramified Iba-1^+^ microglia changed into round Iba-1^+^/CD45^+^ cells, the round Iba-1^+^/CD45^+^ cells could be monocytes infiltrated from the blood.

It has been reported that monocytes/macrophages express two different phenotypes: classically activated and alternatively activated. Classically activated monocytes exert a microbicidal effect through expression of pro-inflammatory mediators, whereas alternatively activated monocytes participate in the repair of damaged tissues through expression of phagocytic activity [34]. Therefore, monocytes could play complex roles in damaged tissues [55]. Accordingly, some studies have reported that monocytes infiltrated into injured brain and spinal cord play neurotoxic roles [56,57], but other studies have reported that monocytes regenerate injured axons and play important roles in recovery after SCI [55,58]. The results in the study suggest that Iba-1^+^/CD45^+^ monocytes and/or microglia detected at 3 and 7 d after SCI function to remove cell debris and repair injured tissues.

Finally, we found that the spatial and temporal patterns of astrocyte and neuronal death were similar (Figures 2 and 5). GFAP and S100β immunoreactivity in P1 ventral regions was detectable at 12 h, but not at 18 h or thereafter (Figures 2 and 5, Additional file 2: Figure S2). mRNA levels of GLT-1 decreased at 3 and 12 h, and those of Kir4.1 decreased at 12 and 24 h (Figure 5B, C). In immunohistochemistry, GLT-1 and Kir4.1 levels decreased in the medial and ventral areas but not in the dorsal areas at 14 h (Figure 5D). At 3 d, GLT-1, and Kir4.1 as well as GFAP and S100β disappeared in P1 ventral regions as in epicenter (Additional file 2: Figure S2A-D). Therefore, loss of the supportive roles of astrocytes could contribute to delayed neuronal death. Astrocytes actively function to maintain brain homeostasis as well as to provide growth factors and nutrients [11-15]. The transporters of glutamate (EAAT1/GLAST and EAAT2/GLT-1) and potassium (Kir4.1) are mainly expressed in astrocytes, and modulate extracellular glutamate and potassium concentration, respectively [17,44,59,60]. Since increase in extracellular glutamate evokes neuronal excitotoxicity [44], abnormal glutamate uptake increases neuronal death [17,44,61]. The latest research has reported that reduced GLT-1 expression aggravated SCI [62]. Dysfunction of glutamate transporters is also associated with neurodegenerative diseases, such as amyotrophic lateral sclerosis (ALS) and Huntington’s disease [63,64]. In addition, increase in extracellular K^+^ also induces neuronal death through calcium influx and excitotoxicity [65]. In the SOD1(G93A) ALS mice, a progressive decrease in Kir4.1 expression was observed in the ventral horn in pre-symptomatic stages [66]. In Kir4.1−/− mice, hair cells and spiral ganglion neurons degenerated [67]. In this study, we found that the spatial and temporal patterns of astrocyte and neuronal death were similar (Figures 2 and 5). Furthermore, mRNA levels of GLT-1 decreased at 3 and 12 h, and those of Kir4.1 decreased at 12 and 24 h (Figure 5B, C). In immunohistochemistry, GLT-1 and Kir4.1 expression decreased in the medial and ventral areas where neuronal death occurred (Figure 5D). The spatial and temporal correlation between neuron and astrocyte damage was also found in ATP-, NMDA- and kainic acid-injected brain cortexes (Unpublished observation). Injection of ATP or NMDA into the brain induced acute damage (3 h) to neurons and astrocytes and did not cause a further increase at later times (3 and/or 7 d). However, in kainic acid-injected brains, neuron and astrocyte damage progressively increased from 3 h to 7 d. Furthermore, in all three injection models, neuron-damage areas corresponded to astrocyte-damage areas (Unpublished observation). Potential support for this interpretation is provided by a recent report that transplantation of human astrocytes facilitates functional recovery after SCI [68]. Thus, we speculate that astrocyte death and/or loss of astrocyte function could be at least a factor in the induction of neuronal death. A potential important focus of future studies is determining whether preservation of astrocytes might protect neurons and prevent delayed neuronal death in SCI.

## **Conclusion**

In SCI, inflammation has been considered as a cause of delayed neuronal damage. However, inflammation appears to function to remove cell debris and repair injured tissues. Thus, loss of supportive function of astrocytes may be critical for delayed neuronal death in SCI.

## **Abbreviations**

ATP, Adenosine-5'-triphosphate; CD45, Cluster of differentiation 45; CD68, Cluster of differentiation 68; CNS, Central nervous system; COX-2, Cyclooxygenase-2; EAAT, Excitatory amino-acid transporters; GAPDH, Glyceraldehyde-3-phosphate dehydrogenase; GFAP, Glial fibrillary acidic protein; GLAST, Glutamate aspartate transporter; GLT-1, Glutamate Transporter 1; GSH, Glutathione; Iba-1, Ionized calcium binding adaptor molecule 1; IL-1β, Interleukin-1beta; IL-6, Interleukin-6; iNOS, Inducible nitric oxide synthase; Kir4.1, Inwardly rectifying potassium channel 4.1; LPS, Lipopolysaccharides; MR, Mannose receptor; NeuN, Neuronal Nuclei; NMDA, N-methyl-D-aspartic acid; ROS, Reactive oxygen species; SCI, Spinal cord injury; TNF-α, Tumor necrosis factor alpha; TUNEL, Terminal deoxynucleotidyl transferase-mediated deoxyuridine triphosphate nick end labeling.

## **Competing interests**

The authors have no financial conflicts of interest.

## **Authors’ contributions**

KM designed the study and performed the bulk of the experiments and analyzed the data, and wrote the manuscript. HJ performed immunohistochemistry and analyzed the data, and wrote the manuscript. BK assisted with the molecular biology experiments and analyzed the data. HS and DH performed part of the animal experiments. ATN and JK assisted with the animal work. IJ provided the materials and equipment for the experiments. EJ supervised the design of the study and coordination, analyzed the data, and wrote the manuscript. BGK assisted with the preparation of the spinal cord injury model and analyzed the data. All authors have read and approved the final version of this manuscript.

## Supplementary Material

Additional file 1 **Figure S1.** Preparation and analysis of cross section of contusion-induced spinal cords. Description: Spinal cords between T7 and T10 were removed. About 300 cross sections (30 μm) from rostral to caudal direction were prepared using a cryostat. (**A**) Sections were attached to 30 to 35 slides as in the first slide, and the second slide was attached to the second slide, and so on. In this way, each slides included 9 to 12 sections. (**B**) Three slides that included immediately adjacent sections obtained at 12 h and 3 d after the injury were stained with antibodies specific for NeuN, GFAP, and lba-1, respectively. Images were taken and arranged from rostral to caudal. Arrowheads at 12 h and 3 d indicate the section where neurons were damaged in the ventral areas. Asterisks(*) at 12 h indicate the section where NeuN^+^ neurons and GFAP^+^ astrocytes were alive but lba-1^+^ microglia died in the ventral areas. Epi, epicenter, P1, penumbra 1; P2, penumbra 2. Scale bar: 500 μm. Click here for file

Additional file 2 **Figure S2.** Time dependent changes in expression of astrocyte markers in SCI. Description: Serial slides including adjacent section were stained with the antibodies specific for GFAP (A), S100β (B; 1:800, Swant Bellinzona, Switzerland), GLT-1 (C), and Kir4. 1 (D). Lower panels are higher magnification image of indicated regions in upper panels. Scale bar; 200 μm (upper panel), 50 μm (lower panel). Click here for file

Additional file 3 **Figure S3.** IL-1β protein was detected in morphologically activated microglia in the P2 area. Description: Sections were obtained from intact and P2 areas at 12h after SCI, stained with IL-1β antibodies, and visualized using HRP-conjugated secondary antibodies. Scale bar: 100 μm and 20 μm (inset). Click here for file

Additional file 4 **Figure S4.** iNOS was detected in neutrophils in injured spinal cords. Description: **(A)** Sections were obtained at the indicated times after SCI and stained with myeloperoxidase (MPO, a neutrophil marker) antibodies and visualized with HRP-conjugated secondary antibodies. **(B)** Sections obtained at 1 d after SCI were stained with MPO/iNOS, and then visualized with Alexa 555 (red)-, and Alexa 488 (green)-conjugated secondary antibodies. Nuclei were counterstained with DAPI. Scale bars: 20 μm. Click here for file

## References

[B1] Taoka Y Okajima K Spinal cord injury in the rat Prog Neurobiol 1998 56 341 358 10.1016/S0301-0082(98)00049-5 9770243

[B2] Hausmann ON Post-traumatic inflammation following spinal cord injury Spinal Cord 2003 41 369 378 10.1038/sj.sc.3101483 12815368

[B3] Nimmerjahn A Kirchhoff F Helmchen F Resting microglial cells are highly dynamic surveillants of brain parenchymain vivo Science 2005 308 1314 1318 10.1126/science.1110647 15831717

[B4] Lee DY Oh YJ Jin BK Thrombin-activated microglia contribute to death of dopaminergic neurons in rat mesencephalic cultures: dual roles of mitogen-activated protein kinase signaling pathways Glia 2005 51 98 110 10.1002/glia.20190 15789435

[B5] Meda L Cassatella MA Szendrei GI Otvos L Baron P Villalba M Ferrari D Rossi F Activation of microglial cells by beta-amyloid protein and interferon-gamma Nature 1995 374 647 650 10.1038/374647a0 7715705

[B6] Chao CC Hu S Molitor TW Shaskan EG Peterson PK Activated microglia mediate neuronal cell injury via a nitric oxide mechanism J Immunol 1992 149 2736 2741 1383325

[B7] Polazzi E Monti B Microglia and neuroprotection: fromin vitrostudies to therapeutic applications Prog Neurobiol 2010 92 293 315 10.1016/j.pneurobio.2010.06.009 20609379

[B8] Wee YV Inflammation in neurological disorders: a help or a hindrance? Neuroscientist 2010 16 408 420 10.1177/1073858410371379 20817918

[B9] Jeong HK Ji KM Kim B Kim J Jou I Joe EH Inflammatory responses are not sufficient to cause delayed neuronal death in ATP-induced acute brain injury PLoS One 2010 5 e13756 10.1371/journal.pone.0013756 21060796 PMC2966428

[B10] Ji KA Yang MS Jeong HK Min KJ Kang SH Jou I Joe EH Resident microglia die and infiltrated neutrophils and monocytes become major inflammatory cells in lipopolysaccharide-injected brain Glia 2007 55 1577 1588 10.1002/glia.20571 17823975

[B11] Badaut J Lasbennes F Magistretti PJ Regli L Aquaporins in brain: distribution, physiology, and pathophysiology J Cereb Blood Flow Metab 2002 22 367 378 11919508 10.1097/00004647-200204000-00001

[B12] Raps SP Lai JC Hertz L Cooper AJ Glutathione is present in high concentrations in cultured astrocytes but not in cultured neurons Brain Res 1989 493 398 401 10.1016/0006-8993(89)91178-5 2765907

[B13] Tsacopoulos M Magistretti PJ Metabolic coupling between glia and neurons J Neurosci 1996 16 877 885 8558256 10.1523/JNEUROSCI.16-03-00877.1996 PMC6578818

[B14] Muller HW Seifert W A neurotrophic factor (NTF) released from primary glial cultures supports survival and fiber outgrowth of cultured hippocampal neurons J Neurosci Res 1982 8 195 204 10.1002/jnr.490080209 7154112

[B15] Gegelashvili G Schousboe A Cellular distribution and kinetic properties of high-affinity glutamate transporters Brain Res Bull 1998 45 233 238 10.1016/S0361-9230(97)00417-6 9510415

[B16] Olsen ML Higashimori H Campbell SL Hablitz JJ Sontheimer H Functional expression of Kir4.1 channels in spinal cord astrocytes Glia 2006 53 516 528 10.1002/glia.20312 16369934 PMC2553202

[B17] Rothstein JD Dykes-Hoberg M Pardo CA Bristol LA Jin L Kuncl RW Kanai Y Hediger MA Wang Y Schielke JP Welty DF Knockout of glutamate transporters reveals a major role for astroglial transport in excitotoxicity and clearance of glutamate Neuron 1996 16 675 686 10.1016/S0896-6273(00)80086-0 8785064

[B18] Ermakova IV Loseva EV Hodges H Sinden J Transplantation of cultured astrocytes attenuates degenerative changes in rats with kainic acid-induced brain damage Bull Exp Biol Med 2005 140 677 681 10.1007/s10517-006-0052-0 16848222

[B19] Myer DJ Gurkoff GG Lee SM Hovda DA Sofroniew MV Essential protective roles of reactive astrocytes in traumatic brain injury Brain 2006 129 2761 2772 10.1093/brain/awl165 16825202

[B20] Bao F John SM Chen Y Mathison RD Weaver LC The tripeptide phenylalanine-(D) glutamate-(D) glycine modulates leukocyte infiltration and oxidative damage in rat injured spinal cord Neuroscience 2006 140 1011 1022 10.1016/j.neuroscience.2006.02.061 16581192

[B21] Matsumoto H Kumon Y Watanabe H Ohnishi T Shudou M Ii C Takahashi H Imai Y Tanaka J Antibodies to CD11b, CD68, and lectin label neutrophils rather than microglia in traumatic and ischemic brain lesions J Neurosci Res 2007 85 994 1009 10.1002/jnr.21198 17265469

[B22] Newman SL Henson JE Henson PM Phagocytosis of senescent neutrophils by human monocyte-derived macrophages and rabbit inflammatory macrophages J Exp Med 1982 156 430 442 10.1084/jem.156.2.430 7097159 PMC2186761

[B23] Yawata I Takeuchi H Doi Y Liang J Mizuno T Suzumura A Macrophage-induced neurotoxicity is mediated by glutamate and attenuated by glutaminase inhibitors and gap junction inhibitors Life Sci 2008 82 1111 1116 10.1016/j.lfs.2008.03.010 18452953

[B24] Koennecke LA Zito MA Proescholdt MG van Rooijen N Heyes MP Depletion of systemic macrophages by liposome-encapsulated clodronate attenuates increases in brain quinolinic acid during CNS-localized and systemic immune activation J Neurochem 1999 73 770 779 10428075 10.1046/j.1471-4159.1999.0730770.x

[B25] Kim HM Hwang DH Choi JY Park CH Suh-Kim H Kim SU Kim BG Differential and cooperative actions of Olig1 and Olig2 transcription factors on immature proliferating cells after contusive spinal cord injury Glia 2011 59 1094 1106 10.1002/glia.21182 21538562

[B26] Kim GM Xu J Xu J Song SK Yan P Ku G Xu XM Hsu CY Tumor necrosis factor receptor deletion reduces nuclear factor-kappaB activation, cellular inhibitor of apoptosis protein 2 expression, and functional recovery after traumatic spinal cord injury J Neurosci 2001 21 6617 6625 11517251 10.1523/JNEUROSCI.21-17-06617.2001 PMC6763083

[B27] Kuhn PL Wrathall JR A mouse model of graded contusive spinal cord injury J Neurotrauma 1998 15 125 140 10.1089/neu.1998.15.125 9512088

[B28] Lehrmann E Kiefer R Christensen T Toyka KV Zimmer J Diemer NH Hartung HP Finsen B Microglia and macrophages are major sources of locally produced transforming growth factor-beta1 after transient middle cerebral artery occlusion in rats Glia 1998 24 437 448 10.1002/(SICI)1098-1136(199812)24:4<437::AID-GLIA9>3.0.CO;2-X 9814824

[B29] Batchelor PE Liberatore GT Wong JY Porritt MJ Frerichs F Donnan GA Howells DW Activated macrophages and microglia induce dopaminergic sprouting in the injured striatum and express brain-derived neurotrophic factor and glial cell line-derived neurotrophic factor J Neurosci 1999 19 1708 1716 10024357 10.1523/JNEUROSCI.19-05-01708.1999 PMC6782182

[B30] Sedgwick JD Schwender S Imrich H Dorries R Butcher GW ter Meulen V Isolation and direct characterization of resident microglial cells from the normal and inflamed central nervous system Proc Natl Acad Sci USA 1991 88 7438 7442 10.1073/pnas.88.16.7438 1651506 PMC52311

[B31] Campanella M Sciorati C Tarozzo G Beltramo M Flow cytometric analysis of inflammatory cells in ischemic rat brain Stroke 2002 33 586 592 10.1161/hs0202.103399 11823674

[B32] Bao F Bailey CS Gurr KR Bailey SI Rosas-Arellano MP Dekaban GA Weaver LC Increased oxidative activity in human blood neutrophils and monocytes after spinal cord injury Exp Neurol 2009 215 308 316 10.1016/j.expneurol.2008.10.022 19056384

[B33] Holness CL Simmons DL Molecular cloning of CD68, a human macrophage marker related to lysosomal glycoproteins Blood 1993 81 1607 1613 7680921

[B34] Martinez FO Helming L Gordon S Alternative activation of macrophages: an immunologic functional perspective Annu Rev Immunol 2009 27 451 483 10.1146/annurev.immunol.021908.132532 19105661

[B35] Dhandapani KM Hadman M De Sevilla L Wade MF Mahesh VB Brann DW Astrocyte protection of neurons: role of transforming growth factor-beta signaling via a c-Jun-AP-1 protective pathway J Biol Chem 2003 278 43329 43339 10.1074/jbc.M305835200 12888549

[B36] Tanaka J Toku K Zhang B Ishihara K Sakanaka M Maeda N Astrocytes prevent neuronal death induced by reactive oxygen and nitrogen species Glia 1999 28 85 96 10.1002/(SICI)1098-1136(199911)28:2<85::AID-GLIA1>3.0.CO;2-Y 10533053

[B37] Huang R Shuaib A Hertz L Glutamate uptake and glutamate content in primary cultures of mouse astrocytes during anoxia, substrate deprivation and simulated ischemia under normothermic and hypothermic conditions Brain Res 1993 618 346 351 10.1016/0006-8993(93)91289-5 8104087

[B38] Hertz L An intense potassium uptake into astrocytes, its further enhancement by high concentrations of potassium, and its possible involvement in potassium homeostasis at the cellular level Brain Res 1978 145 202 208 10.1016/0006-8993(78)90812-0 638779

[B39] Min KJ Yang MS Kim SU Jou I Joe EH Astrocytes induce hemeoxygenase-1 expression in microglia: a feasible mechanism for preventing excessive brain inflammation J Neurosci 2006 26 1880 1887 10.1523/JNEUROSCI.3696-05.2006 16467537 PMC6793633

[B40] Kim JH Min KJ Seol W Jou I Joe EH Astrocytes in injury states rapidly produce anti-inflammatory factors and attenuate microglial inflammatory responses J Neurochem 2010 115 1161 1171 10.1111/j.1471-4159.2010.07004.x 21039520

[B41] Liu D Thangnipon W McAdoo DJ Excitatory amino acids rise to toxic levels upon impact injury to the rat spinal cord Brain Res 1991 547 344 348 10.1016/0006-8993(91)90984-4 1884213

[B42] Farooque M Hillered L Holtz A Olsson Y Changes of extracellular levels of amino acids after graded compression trauma to the spinal cord: an experimental study in the rat using microdialysis J Neurotrauma 1996 13 537 548 10.1089/neu.1996.13.537 8913970

[B43] Young W Koreh I Yen V Lindsay A Effect of sympathectomy on extracellular potassium ionic activity and blood flow in experimental spinal cord contusion Brain Res 1982 253 115 124 10.1016/0006-8993(82)90678-3 6295547

[B44] Choi DW Maulucci-Gedde M Kriegstein AR Glutamate neurotoxicity in cortical cell culture J Neurosci 1987 7 357 368 2880937 10.1523/JNEUROSCI.07-02-00357.1987 PMC6568898

[B45] Takahashi S Shibata M Fukuuchi Y Role of sodium ion influx in depolarization-induced neuronal cell death by high KCI or veratridine Eur J Pharmacol 1999 372 297 304 10.1016/S0014-2999(99)00208-3 10395025

[B46] Speake T Whitwell C Kajita H Majid A Brown PD Mechanisms of CSF secretion by the choroid plexus Microsc Res Tech 2001 52 49 59 10.1002/1097-0029(20010101)52:1<49::AID-JEMT7>3.0.CO;2-C 11135448

[B47] Elkabes S Cicco-Bloom EM Black IB Brain microglia/macrophages express neurotrophins that selectively regulate microglial proliferation and function J Neurosci 1996 16 2508 2521 8786427 10.1523/JNEUROSCI.16-08-02508.1996 PMC6578768

[B48] Streit WJ Microglia as neuroprotective, immunocompetent cells of the CNS Glia 2002 40 133 139 10.1002/glia.10154 12379901

[B49] Streit WJ Microglia and neuroprotection: implications for Alzheimer's disease Brain Res Brain Res Rev 2005 48 234 239 15850662 10.1016/j.brainresrev.2004.12.013

[B50] Pineau I Lacroix S Proinflammatory cytokine synthesis in the injured mouse spinal cord: multiphasic expression pattern and identification of the cell types involved J Comp Neurol 2007 500 267 285 10.1002/cne.21149 17111361

[B51] Hamada Y Ikata T Katoh S Tsuchiya K Niwa M Tsutsumishita Y Fukuzawa K Roles of nitric oxide in compression injury of rat spinal cord Free Radic Biol Med 1996 20 1 9 10.1016/0891-5849(95)02017-9 8903674

[B52] Adachi K Yimin Y Satake K Matsuyama Y Ishiguro N Sawada M Hirata Y Kiuchi K Localization of cyclooxygenase-2 induced following traumatic spinal cord injury Neurosci Res 2005 51 73 80 10.1016/j.neures.2004.10.007 15596243

[B53] Bolanos JP Almeida A Roles of nitric oxide in brain hypoxia-ischemia Biochim Biophys Acta 1999 1411 415 436 10.1016/S0005-2728(99)00030-4 10320673

[B54] Fleming JC Norenberg MD Ramsay DA Dekaban GA Marcillo AE Saenz AD Pasquale-Styles M Dietrich WD Weaver LC The cellular inflammatory response in human spinal cords after injury Brain 2006 129 3249 3269 10.1093/brain/awl296 17071951

[B55] Benowitz LI Popovich PG Inflammation and axon regeneration Curr Opin Neurol 2011 24 577 583 10.1097/WCO.0b013e32834c208d 21968547

[B56] Lee SM Rosen S Weinstein P Van Rooijen N Noble-Haeusslein LJ Prevention of both neutrophil and monocyte recruitment promotes recovery after spinal cord injury J Neurotrauma 2011 28 1893 1907 10.1089/neu.2011.1860 21657851 PMC3172879

[B57] Pineau I Sun L Bastien D Lacroix S Astrocytes initiate inflammation in the injured mouse spinal cord by promoting the entry of neutrophils and inflammatory monocytes in an IL-1 receptor/MyD88-dependent fashion Brain Behav Immun 2010 24 540 553 10.1016/j.bbi.2009.11.007 19932745

[B58] Shechter R London A Varol C Raposo C Cusimano M Yovel G Rolls A Mack M Pluchino S Martino G Jung S Schwartz M Infiltrating blood-derived macrophages are vital cells playing an anti-inflammatory role in recovery from spinal cord injury in mice PLoS Med 2009 6 e1000113 10.1371/journal.pmed.1000113 19636355 PMC2707628

[B59] Lehre KP Levy LM Ottersen OP Storm-Mathisen J Danbolt NC Differential expression of two glial glutamate transporters in the rat brain: quantitative and immunocytochemical observations J Neurosci 1995 15 1835 1853 7891138 10.1523/JNEUROSCI.15-03-01835.1995 PMC6578153

[B60] Danbolt NC Glutamate uptake Prog Neurobiol 2001 65 1 105 10.1016/S0301-0082(00)00067-8 11369436

[B61] Watase K Hashimoto K Kano M Yamada K Watanabe M Inoue Y Okuyama S Sakagawa T Ogawa S Kawashima N Hori S Takimoto M Wada K Tanaka K Motor discoordination and increased susceptibility to cerebellar injury in GLAST mutant mice Eur J Neurosci 1998 10 976 988 10.1046/j.1460-9568.1998.00108.x 9753165

[B62] Lepore AC O'Donnell J Kim AS Yang EJ Tuteja A Haidet-Phillips A O'Banion CP Maragakis NJ Reduction in expression of the astrocyte glutamate transporter, GLT1, worsens functional and histological outcomes following traumatic spinal cord injury Glia 2011 59 1996 2005 10.1002/glia.21241 21882244 PMC3269541

[B63] Liévens JC Woodman B Mahal A Spasic-Boscovic O Samuel D Kerkerian-Le Goff L Bates GP Impaired glutamate uptake in the R6 Huntington's disease transgenic mice Neurobiol Dis 2001 8 807 821 10.1006/nbdi.2001.0430 11592850

[B64] Arzberger T Krampfl K Leimgruber S Weindl A Changes of NMDA receptor subunit (NR1, NR2B) and glutamate transporter (GLT1) mRNA expression in Huntington's disease–anin situhybridization study J Neuropathol Exp Neurol 1997 56 440 454 10.1097/00005072-199704000-00013 9100675

[B65] Choi DW Ionic dependence of glutamate neurotoxicity J Neurosci 1987 7 369 379 2880938 10.1523/JNEUROSCI.07-02-00369.1987 PMC6568907

[B66] Kaiser M Maletzki I Hulsmann S Holtmann B Schulz-Schaeffer W Kirchhoff F Bahr M Neusch C Progressive loss of a glial potassium channel (KCNJ10) in the spinal cord of the SOD1 (G93A) transgenic mouse model of amyotrophic lateral sclerosis J Neurochem 2006 99 900 912 10.1111/j.1471-4159.2006.04131.x 16925593

[B67] Rozengurt N Lopez I Chiu CS Kofuji P Lester HA Neusch C Time course of inner ear degeneration and deafness in mice lacking the Kir4.1 potassium channel subunit Hear Res 2003 177 71 80 10.1016/S0378-5955(02)00799-2 12618319

[B68] Davies SJ Shih CH Noble M Mayer-Proschel M Davies JE Proschel C Transplantation of specific human astrocytes promotes functional recovery after spinal cord injury PLoS One 2011 6 e17328 10.1371/journal.pone.0017328 21407803 PMC3047562

