# Supplementary figures and images for: Spatial and temporal correlation in progressive degeneration of neurons and astrocytes in contusion-induced spinal cord injury
Source: J Neuroinflammation. 2012 May 25;9:100. doi: 10.1186/1742-2094-9-100 (PMC3418552; doi:10.1186/1742-2094-9-100)

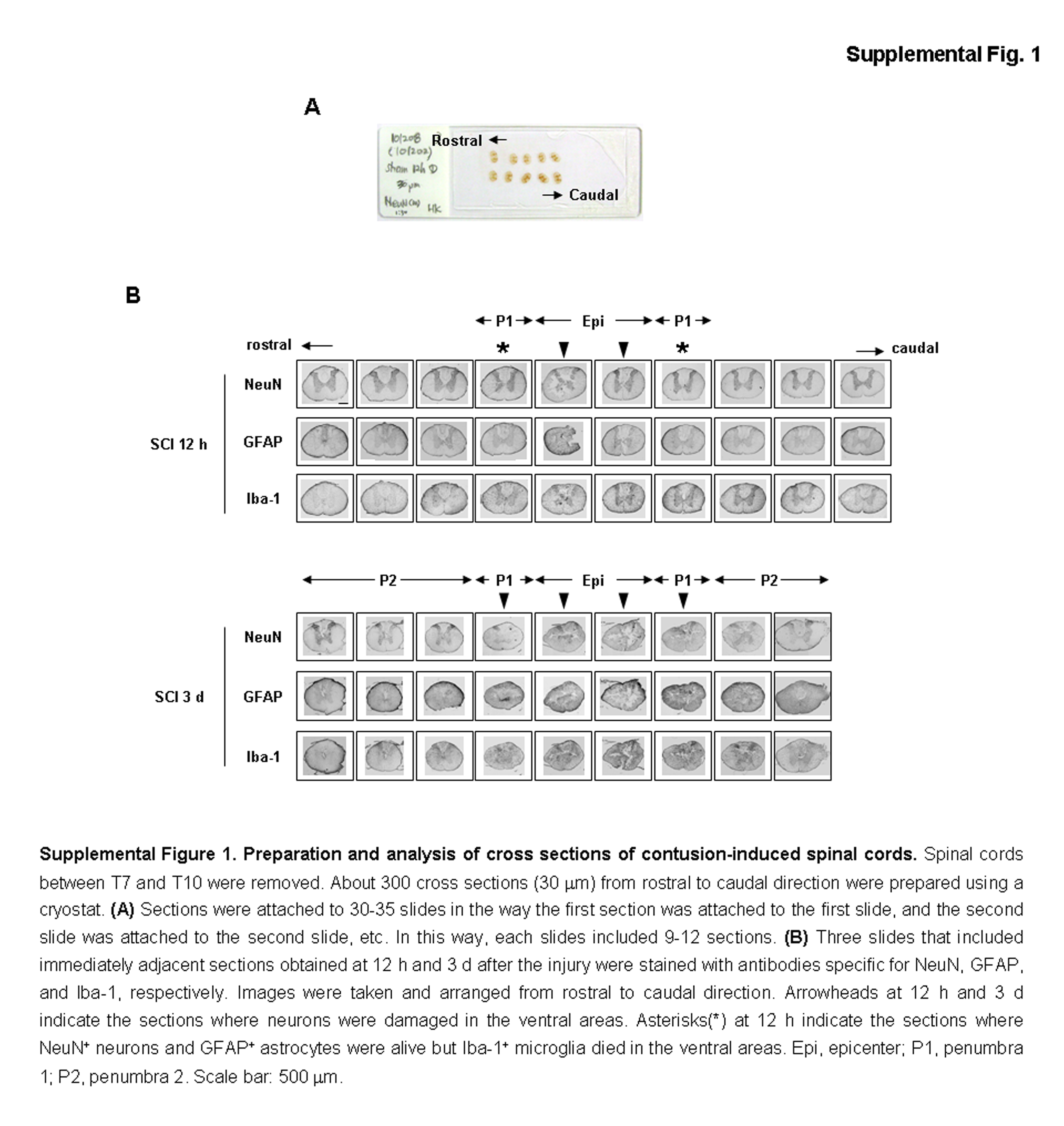

Supplement: Additional file 1 — Figure S1. Preparation and analysis of cross section of contusion-induced spinal cords. Description: Spinal cords between T7 and T10 were removed. About 300 cross sections (30 μm) from rostral to caudal direction were prepared using a cryostat. (A) Sections were attached to 30 to 35 slides as in the first slide, and the second slide was attached to the second slide, and so on. In this way, each slides included 9 to 12 sections. (B) Three slides that included immediately adjacent sections obtained at 12 h and 3 d after the injury were stained with antibodies specific for NeuN, GFAP, and lba-1, respectively. Images were taken and arranged from rostral to caudal. Arrowheads at 12 h and 3 d indicate the section where neurons were damaged in the ventral areas. Asterisks(*) at 12 h indicate the section where NeuN+ neurons and GFAP+ astrocytes were alive but lba-1+ microglia died in the ventral areas. Epi, epicenter, P1, penumbra 1; P2, penumbra 2. Scale bar: 500 μm. [file 1742-2094-9-100-S1.tiff]

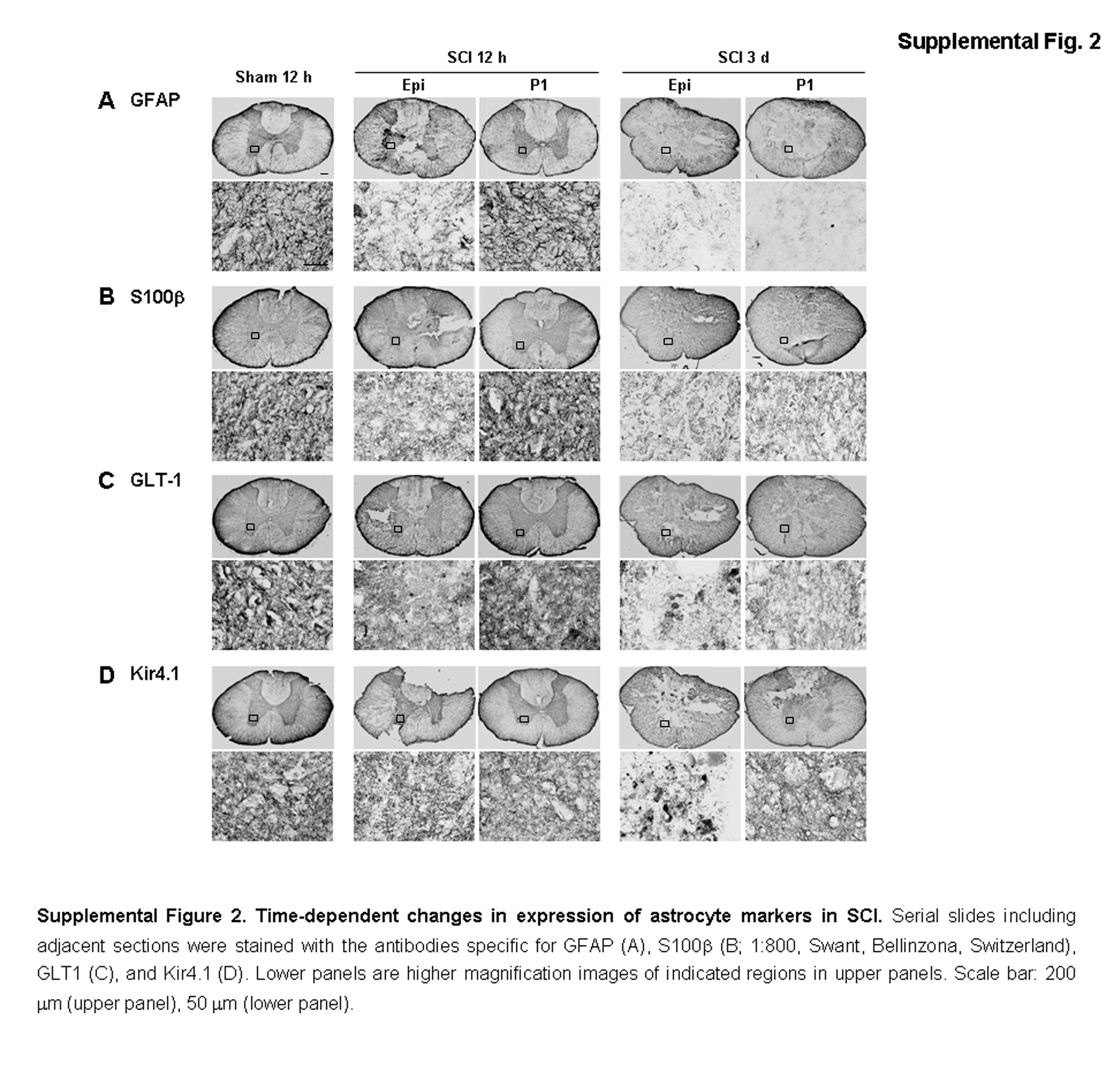

Supplement: Additional file 2 — Figure S2. Time dependent changes in expression of astrocyte markers in SCI. Description: Serial slides including adjacent section were stained with the antibodies specific for GFAP (A), S100β (B; 1:800, Swant Bellinzona, Switzerland), GLT-1 (C), and Kir4. 1 (D). Lower panels are higher magnification image of indicated regions in upper panels. Scale bar; 200 μm (upper panel), 50 μm (lower panel). [file 1742-2094-9-100-S2.tiff]

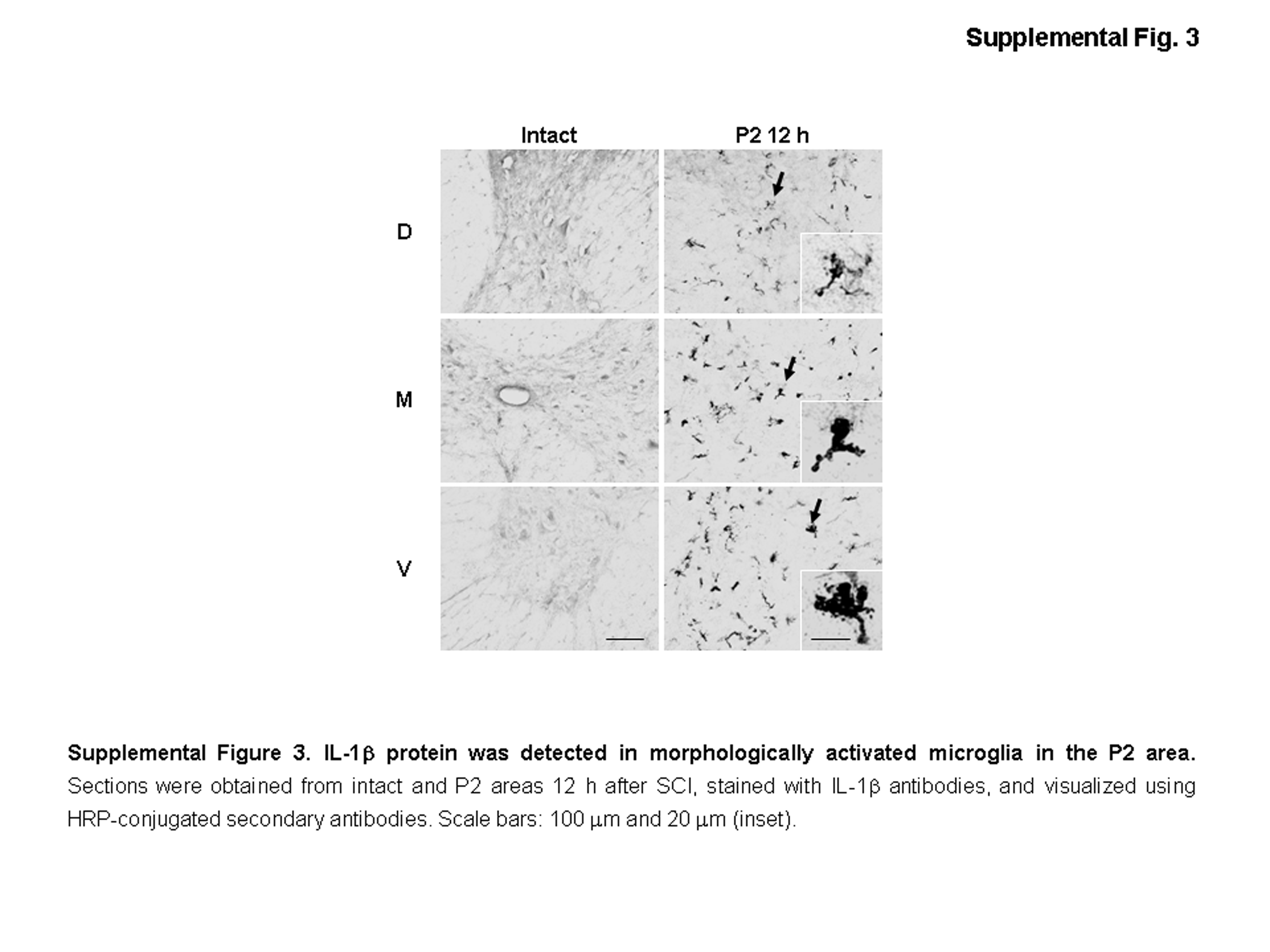

Supplement: Additional file 3 — Figure S3. IL-1β protein was detected in morphologically activated microglia in the P2 area. Description: Sections were obtained from intact and P2 areas at 12h after SCI, stained with IL-1β antibodies, and visualized using HRP-conjugated secondary antibodies. Scale bar: 100 μm and 20 μm (inset). [file 1742-2094-9-100-S3.tiff]

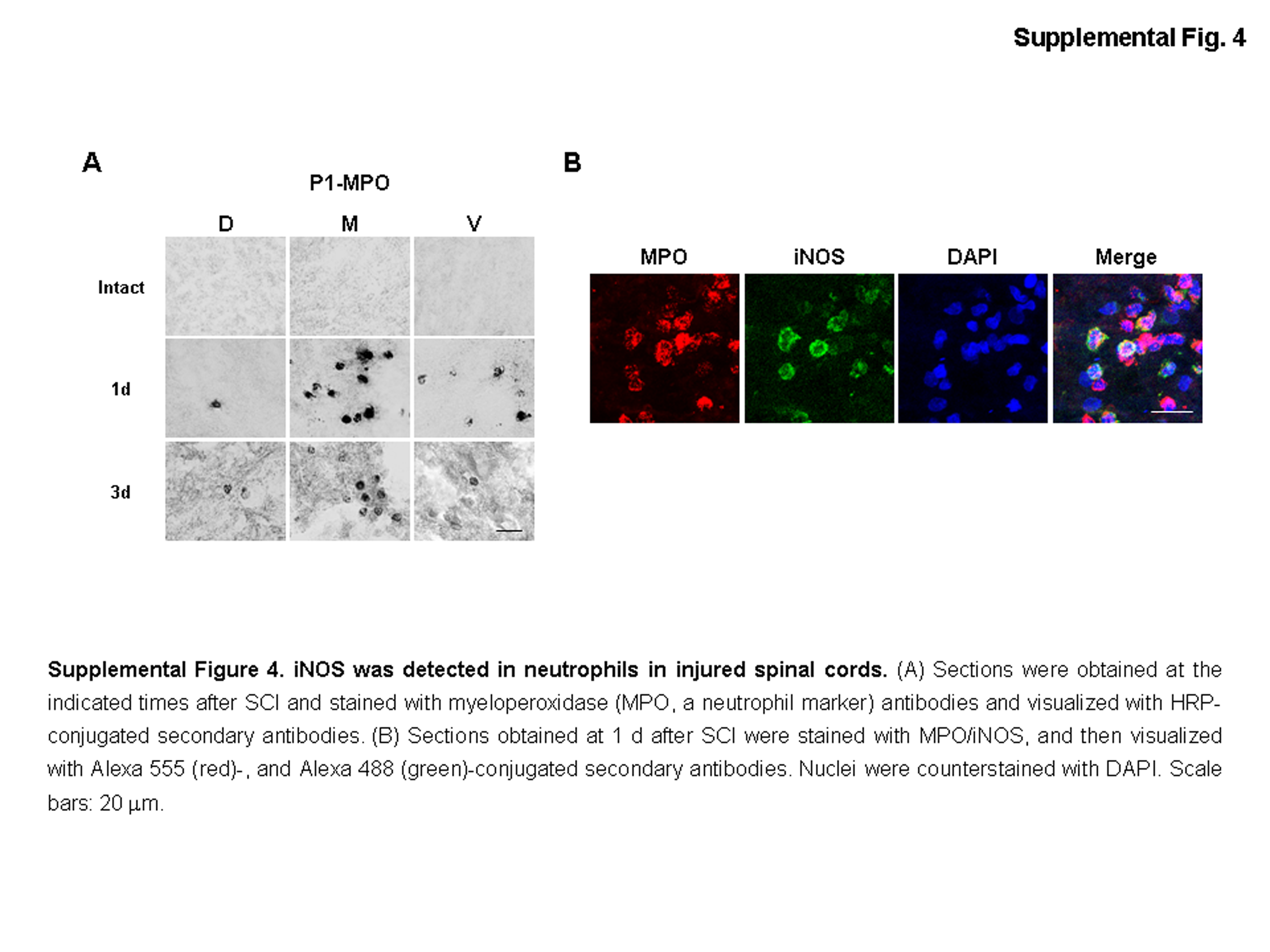

Supplement: Additional file 4 — Figure S4. iNOS was detected in neutrophils in injured spinal cords. Description: (A) Sections were obtained at the indicated times after SCI and stained with myeloperoxidase (MPO, a neutrophil marker) antibodies and visualized with HRP-conjugated secondary antibodies. (B) Sections obtained at 1 d after SCI were stained with MPO/iNOS, and then visualized with Alexa 555 (red)-, and Alexa 488 (green)-conjugated secondary antibodies. Nuclei were counterstained with DAPI. Scale bars: 20 μm. [file 1742-2094-9-100-S4.tiff]
